# Early Complications of Ventriculoperitoneal Shunt in Pediatric Patients With Hydrocephalus

**DOI:** 10.7759/cureus.13506

**Published:** 2021-02-23

**Authors:** Bilal Khan, Saima Hamayun, Usman Haqqani, Khalid Khanzada, Sajjad Ullah, Rizwanullah Khattak, Nasrullah Zadran, Zohra Bibi, Abdul Wali Khan

**Affiliations:** 1 Neurosurgery, Lady Reading Hospital-Medical Teaching Institutions (MTI), Peshawar, PAK; 2 Medicine, Khyber Medical College, Peshawar, PAK; 3 Neurosurgery, Qazi Hussain Ahmed Medical Complex, Nowshera, PAK; 4 Neurosurgery, Ibrahimi Medical Center, Peshawar, PAK; 5 Neurosurgery, Khyber Teaching Hospital-Medical Teaching Institutions (MTI), Peshawar, PAK; 6 Neurosurgery, Hayatabad Medical Complex-Medical Teaching Institutions (MTI), Peshawar, PAK; 7 Internal Medicine, Mayo Hospital, Lahore, PAK; 8 Psychiatry, Lady Reading Hospital-Medical Teaching Institutions (MTI), Peshawar, PAK; 9 Internal Medicine, College of Physicians and Surgeons Pakistan, Peshawar, PAK; 10 Internal Medicine, Hayatabad Medical Complex Peshawar, Peshawar, PAK

**Keywords:** complications, shunt, ventriculoperitoneal(vp), early, infection, csf leak

## Abstract

Objective

Ventriculoperitoneal shunt (VPS) is the most commonly used procedure for the treatment of hydrocephalus (HDC), especially in children. However, this is prone to many complications, and requires repeated surgeries, which as such increases the morbidity of the patients. It is estimated that majority of the complications occurs in the immediate post-operative period and the rate of complications decreases over the time, with no impunity to these, though. We conducted this study to know about the complications of VPS in the early post-operative period, in pediatric patients with hydrocephalus.

Materials and methods

This descriptive study was conducted in the Department of Neurosurgery, Lady Reading Hospital, Peshawar, between June 2019 and January 2020 (seven months). All patients with hydrocephalus below 12 years of age, operated for the first time were included after taking an informed consent, while those with repeated shunt procedures and elderly patients requiring shunt were excluded from the study. Patients’ details like age, gender, location, contact number, cause of hydrocephalus, date of shunt placement, type of surgery (elective or emergency) and any follow-up complications like failure, erosion, infections, ileus were noted on a predesigned proforma. After the surgery, patients were followed for a period of one month, and contacted either through the telephone or asked to visit in the outpatients on the specified days, and were evaluated for any shunt-related complications, and any of these complications suspected were further evaluated and noted. The data was analyzed using the statistical program SPSS version 19 (IBM Corp., Armonk, NY). Results are presented in the form of charts, tables and graph.

Results

During the study period, we evaluated a total of 151 patients; there were 78 (51.65%) males and 73 (48.34%) females with a male to female ratio approaching 1.1:1. The age range was from 22 days to 12 years. The mean age was 38.46 ± 7.53 months. The primary indications for the insertion of VP shunt were: congenital hydrocephalus in 70 (46.4%), post infectious hydrocephalus in 57 (37.7%), hydrocephalus due to tumor in 22 (14.6%), and post traumatic hydrocephalus in two (1.4%) patients. Among the total number of patients, 85 patients (55.6%) were done as elective cases and 66 patients (44.4%) were done as emergency cases. Complications were encountered in 30 patients (19.87%) during the follow-up of 30 days. Complications occurred between day 1 and day 20 of follow-up with a mean of 9.10 ± 1.69 2SD days.

Conclusion

VP shunt is the most widely used treatment for HDC, but is predisposed to complications and almost every fifth case of VP shunt comes across with complications. Shunt blockage, infections and abdominal wound-related complications are common earlier complications in pediatric patients with hydrocephalus.

## Introduction

Ventriculoperitoneal shunt (VPS) is a common procedure performed for the treatment of hydrocephalus (HDC), which is a brain condition due to excessive production or decrease absorption of cerebrospinal fluid (CSF) resulting in dilatation of the ventricular system [1, 2]. It is a common condition accounting for 69,000 hospital discharges each year in the United States and there are approximately 36,000 CSF shunt procedures performed annually, amounting to $94 million of medical costs per year [1, 2]. A ventriculoperitoneal (VP) shunt is a CSF diversion device, usually a tube, with a pressure-regulating valve that begins in the ventricular system and carries CSF to an absorptive surface outside the brain such as the peritoneum [2]. However, there can be other shunt systems such as ventriculopleural or ventriculoatrial shunts carrying it to the pleura and the vascular system, respectively [2,3], and can be treated at times even without the use of shunt [4], but in 98% of cases it is the peritoneal cavity [2-4]. The aim of VP shunting is to relieve excess intra cranial pressure (ICP) due to HDC [1-3, 5].

The major complications of VP shunt are mechanical malfunction, placement failure, infection, CSF leak [3,5-7] with some reports of even intracerebral haemorrhage, though rare, and complications related with peritoneal catheter such as ileus, pseudocyst formation, and bowel perforation, resulting in shunt failure [3,5,6,8,9]. In pediatric surgical series, shunt failures occur in 14% of patients just within the first month after shunt placement, and it is estimated 4-50% of shunts will fail within the first year [3]. Adults also experience a relatively high (29%) shunt failure rate within the first year. Long-term studies suggest that 45 to 59% of all patients, regardless of age, will require a shunt revision. Multiple shunt failure and infections are common, and revisions account for 48% of all shunt-related procedures [3,6,7].

As stated above, VP shunt is one of the common procedures performed in the neurosurgical department, and pediatrics patients comprise a major part of all these surgeries, and they are more vulnerable to the complications [3,6,9-12], since majority of the complications are reported in the early days of placement of a VP shunt [3,6,11,12], we conducted this study to know about the complications of VP shunt in the first month of placement, in the pediatric population, operated at our department.

## Materials and methods

This descriptive study was conducted in the Department of Neurosurgery, Lady Reading Hospital, Peshawar, between June 2019 and January 2020 (seven months). All patients with hydrocephalus below 12 years of age, operated for the first time were included after taking an informed consent and sampling was done through consecutive non-probability sampling, while those with repeated shunt procedures, elderly patients requiring shunt, those expired during the study period and those lost to follow up were excluded from the study. Patients’ details like age, gender, location, contact number, cause of hydrocephalus, date of shunt placement, type of surgery (elective or emergency) and any follow-up complications like failure, erosion, infections, ileus were noted on a predesigned proforma. After the surgery, patients were followed for a period of one month, and contacted either through the telephone or asked to visit to the outpatients on the specified days, and were evaluated for any shunt-related complications, and any of these complications suspected were further evaluated and noted. The data was analyzed using the statistical program SPSS version 19 (IBM Corp., Armonk, NY). Results are presented in the form of charts, tables and graph.

## Results

During the study period, we evaluated a total of 151 patients; there were 78 (51.65%) males and 73 (48.34%) females with a male to female ratio approaching 1.1:1. The age range was from 22 days to 12 years. The mean age was 38.46 ± 7.53 months. The minimum age was 22 days and the maximum age was 12 years. There were 72 (47.7%) patients below one year of age representing the majority, and 45 (29.8%) patients were in the age group of 1-6 years, while there were 34 (22.5%) patients between six and 12 years of age. Regarding the primary indications for the insertion of VP shunt there were congenital in 70 (46.4%) patients in the form of aqueductal stenosis, patients with myelomeningocele and Dandy-Walker syndrome, intracranial bleed. There were 57 (37.7%) post infectious cases including post meningitic and tuberculous meningitis (shunt was placed after a clear CSF with no neutrophils and decreased proteins), and hydrocephalus due to tumor was found in 22 (14.6%) patients. Two patients (1.4%) were having hydrocephalus related to the trauma, i.e., post traumatic hydrocephalus; all are summarized in Table 1.

**Table 1:** The indications for placement of the ventriculoperitoneal (VP) shunt.

	Indications	Frequency	Percent	Valid Percent	Cumulative Percent
Valid	Congenital	70	46.4	46.4	46.4
	Infectious	57	37.7	37.7	84.1
	Tumor	22	14.6	14.6	98.7
	Post trauma	2	1.3	1.3	100.0
	Total	151	100.0	100.0

The procedures were performed either as elective cases or as an emergency. Among the total number of patients, 85 patients (55.6%) were done as elective cases and 66 (44.4%) patients were done as emergency cases. The operations were done either by the senior residents or by consultants. Patients were followed for one month regarding any complications like shunt malfunction, infections, CSF leaks, seizures, ileus and complications germane to the peritoneal catheter like exposure of the distal catheter, extrusion through the anus, and/or placement of the catheter outside the peritoneal cavity. Complications were encountered in 30 patients (19.87%) during the follow-up of 30 days. Shunt malfunction due to the blockage of the ventricular catheter was present in 11 (7.3%), infection in seven (4.6%), CSF leak in one (0.7%), ileus was in two (1.3%), seizures were in three (2.0%) and complications pertinent to the abdominal catheter were present in six (4.0%) patients as shown in Table 2.

**Table 2:** Frequency of various complications related to ventriculoperitoneal (VP) shunt in pediatric patients. CSF: Cerebrospinal fluid

	Complications	Frequency	Percent	Valid Percent	Cumulative Percent
Valid	Congenital	121	80.1	80.1	80.1
	Malfunction	11	7.3	7.3	87.4
	Infection	7	4.6	4.6	92.1
	CSF leak	1	.7	.7	92.7
	Ileus	2	1.3	1.3	94.0
	Seizures	3	2.0	2.0	96.0
	Abdominal	6	4.0	4.0	100.0
	Total	151	100.0	100.0

**Figure 1:**
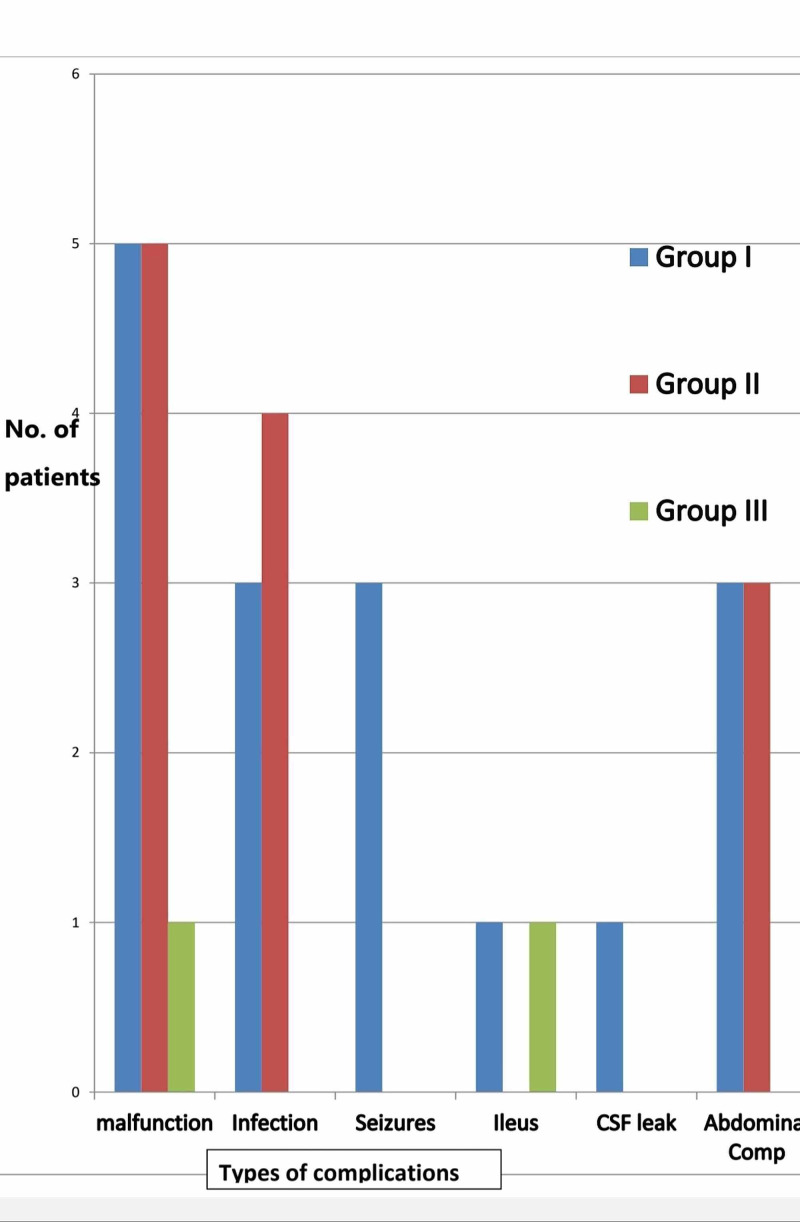
The distribution of documented complications among different age groups. Group I (<1 year) harbors the majority of complications followed by the Group II (1-5 years) while the complications are minimal in Group III (6-12 years).

Complications occurred between day 1 and day 20 of follow-up with a mean of 9.10 ± 1.69 2SD days. Complications like seizures and ileus were evident early whereas infections, malfunctions and peritoneal catheter-related complications revealed later. Complications were stratified among the age, gender, indications for surgery and the timing of surgery, i.e., whether as an elective or emergency case. The patients in age group I (72 patients) were having 16 (11.25%) complications; there were 12 (7.2%) complications out of the 45 patients in age group II and two (1.4%) complications occurred in the age group III among 34 patients, as shown in Figure 1.

Gender-wise stratification of the complications showed that out of the 30 patients with complications, 17 (11.26%) were males and 13 (8.6%) were females. Infections were more common in females (5/7 patients) while abdominal complications were more prevalent in males (5/6 patients) as shown in Table 3. Stratification of the complication among the indications for surgery revealed that 17 patients (11.2%) were operated for the congenital hydrocephalus, 10 patients (6.62%) were operated for the infectious causes and three (1.99%) patients were operated for tumor-related pathology. To see whether the complications were more common in elective or the emergency cases stratification was done between these two which showed that the complications were more in patients operated on elective list (18, 11.92%) compared to those on emergency (12 patients, 7.95%). Abdominal complications were more common in patients operated on elective list (4/6 patients) while infection was slightly raised in patients operated as emergency (4/7 patients).

**Table 3:** The various complications and their gender-wise distribution. CSF: Cerebrospinal fluid

			Any/number of complications							Total
			Congenital	Malfunction	Infection	CSF leak	Ileus	Seizures	Abdominal complications	
Gender of patient	Male	Count	61	5	2	1	1	3	5	78
		% within any complication	50.4%	45.5%	28.6%	100.0%	50.0%	100.0%	83.3%	51.7%
	Female	Count	60	6	5	0	1	0	1	73
		% within any complication	49.6%	54.5%	71.4%	0%	50.0%	0%	16.7%	48.3%
		Total Count	121	11	7	1	2	3	6	151
		Total % within any complication	100.0%	100.0%	100.0%	100.0%	100.0%	100.0%	100.0%	100.0%

## Discussion

Hydrocephalus is a major neurosurgical problem encountered by a neurosurgeon. VP shunt is most frequently used procedure for the treatment of hydrocephalus, though endoscopy is also of use in hydrocephalus (HDC) caused by multiple pathologies [2-4,9-11]. However, VP shunt still accounts for more than 70% of the diversion procedures and its use is further high in developing countries like ours, where infectious pathologies, affecting the young are common and endoscopy is not a good option in them [11,12]. But the use of this procedure is associated with a lot of problems and complications {5,6,8-10,13,14].

In our study there was a slight male preponderance over the females. Although hydrocephalus does not have an association with the gender and neither does a particular gender predispose to the hydrocephalus, but several studies show that there is male gender predilection for some unknown reasons [9,11-15]. The age range at the insertion of the VP shunt has been from 20 days to 12 years. Twelve years was taken as the maximum age limit for these patients because of the various cutoff points for pediatric population. Though many studies have taken 18 years as the upper age limit for pediatrics [13,14], in our set up children up to the age of 12 are mostly treated in Pediatrics and are usually accompanied by their mother or chaperone, and usually children above 12 years are treated as grown, usually treated in the wards not specified for pediatrics disorders. Most of the patients in our study group were below 1 year of age (47%). And since many studies have reported complications in the younger age groups as compared to the older age group [3-6,11-13], we further stratified the age into below one year, 1-6 years and 6-12 years to know about the most affected population group in our series. As reported by Cambrin et al., age less than six months accounted for the most in his series, i.e. 55%. He further elaborated the use of endoscope as an independent variable for the shunt failure [13], though we did not study that.

Etiologically, hydrocephalus was classified as either congenital including Dandy-Walker Syndrome (DWS), myelomeningocele (MMC), secondary to intraventricular haemorrhage (IVH), aqueductal stenosis (AS), any other congenital cyst anomaly leading to hydrocephalus, or post meningitic and tumor-related as shown in Table 4.

**Table 4:** The frequency of various causes of HDC as reported and a comparison with our data. HDC: Hydrocephalus

Serial no.	Etiology of Hydrocephalus	Our Study	Reported
1.	Congenital	46.7%	80.1% (Cambrin et al. [13])
			88% (McGirt et al. [12])
2.	Post-infectious	37.4%	3.7% (Cambrin et al. [13])
			40% (Kingsly et al. [9])
3.	Tumor Related	14.6%	18.5% (Cambrin et al. [13])
			13% (McGirt et al. [12])
4.	Post Trauma	1.4%	4.7% (Cambrin et al. [13])

As shown the most common etiology in the developed world has been the congenital hydrocephalus, whereas in the developing world it has been the post-infectious - reported to a very high level. Our study showed congenital and infectious etiologies to be the most common causes.

Despite lot of advances in other areas of medicine, rates of shunt-related complications have been static and ranges to about 30%, with overall long-term shunt failure rate as high as 46% [15,16-18]. On the contrary, we encountered overall complication rate of 19.8% cases (n = 30) only, but this reflects a complication rate at one month and is expected to be higher with long-term follow-ups.

Overall infection rate varies from 3-12%, and is found to be higher in infants than adults [5-7]. Infection may occur any time from days to months with a median at one month [19,20]. We documented a 4.6% infection rate in given period which is comparable to studies conducted in developed countries [21]. Shunt malfunction (Note: in the definition section, malfunction should be defined as non-infectious entity comprising of shunt blockage, shunt breakage or dislodgment), which was noticed in 7.3% cases (n = 11), is still the most common complication of shunt insertion and is the main reason for shunt reinsertion [3,20-22].

A study by Ghritlaharey et al. [22] reported the incidence of CSF leak to be 1.7%, and complications related to abdominal catheter as 13% (18/236 patients) whereas in our study, we observed only one case (0.7% incidence) of CSF leak and complications related to abdominal catheter as 4% (n = 6) which is significantly lower than the previous study. We also observed newly developed seizures after shunt placement in 2% patients (n = 3). Seizures remain to be one of the important problems in patients who undergo VP shunt, 31% to 69% of shunted patients have history of seizures, in addition shunt dysfunction itself predisposes the patient to seizures [23]. Electroencephalogram (EEG) changes including focal specific paroxysmal discharges accompanied by slow waves in ventricular catheter area have been observed and are considered to be associated with the cortical injury in the process of shunt placement [23,24].

Age is important factor influencing shunt survival [12-14,16-18]. We divided our study population in three groups, age group I consisting of patients of less than one year was the most prone to develop complications at the rate of 11.25% (n = 17) followed by 7.2% (n = 11) complication rate for age group II (1-6 year). Children above six years in age group III had a lower complication rate, which is consistent with previous studies performed by Reddy et al. [15], though Davis et al. found no significant difference in the shunt survival based on age in the infants [17]. Though there are several studies that suggest no significant difference in complications on the basis of gender [3,5,24-26], we found complications in male patients to be more, i.e. 11.26% (n = 17), while in females it was 8.6% (n = 13). On further stratification of complications, it was noted that infection was more common in female patients (5/7 patients) while abdominal complications were more prevalent in male patients (5/6 patients), but it was not of any statistical significance.

Studies have shown a significant relation between etiology of hydrocephalus and shunt-associated complications [3,7, 17-23]. In our study, we also found a significant relation, with congenital hydrocephalus being the highest in shunt-related complications (11.2%), followed by infectious cause of hydrocephalus (6.62%), while tumor-related pathology was the least with 1.99%.

Results were also analyzed and stratified on the basis of whether it was done as an elective case or as an emergency case. In the course of our study, out of the total of 151 patients, 55.6% (n = 85) underwent elective surgery while emergency shunting in ER theater had to be undertaken in 44.4% (n = 66). Patients were divided into two groups because most of the cases were done in elective theater by consultants whereas, only patients who were at immediate risk of reduced conscious level or had persistent vomiting with documented signs of hydrocephalous on radiological report were shifted and operated in emergency theater. Surgeries in emergency theater were done by residents. Though there is no significant data available in this regard but there was an impression of increased rate of infection in patients done on emergency basis [19-21, 25,26]. The reason of high infection rate in VP shunt procedures done on emergency basis (11.92% cases) as compared to elective operating room table (7.95% cases) is probably due to trauma, road traffic accidents (RTA) and infected cases done very often in this setting. Anderson et al. showed that the complications were more (22%) in the children when no consultant was involved compared to 12%, when a consultant performed the procedure, but it was not statistically significant [27].

## Conclusions

VP shunt is the most widely used treatment for HDC, but is predisposed to complications, and almost every fifth case of VP shunt comes across with complications. Shunt blockage, infections and abdominal wound-related complications are common earlier complications in pediatric patients with hydrocephalus, requiring shunt revisions. Other less common complications are the CSF leak, seizure, and ileus that may complicate a VPS procedure in the early post-operative period. Age has some implications in increasing the risk of these and the majority of children requiring these procedures are low age.
